# Differences in Mucosal Gene Expression in the Colon of Two Inbred Mouse Strains after Colonization with Commensal Gut Bacteria

**DOI:** 10.1371/journal.pone.0072317

**Published:** 2013-08-09

**Authors:** Frances Brodziak, Caroline Meharg, Michael Blaut, Gunnar Loh

**Affiliations:** 1 Department of Gastrointestinal Microbiology, German Institute of Human Nutrition Potsdam-Rehbruecke, Nuthetal, Germany; 2 Institute for Global Food Security, Queen’s University, Belfast, Northern Ireland; Charité, Campus Benjamin Franklin, Germany

## Abstract

The host genotype has been proposed to contribute to individually composed bacterial communities in the gut. To provide deeper insight into interactions between gut bacteria and host, we associated germ-free C3H and C57BL/10 mice with intestinal bacteria from a C57BL/10 donor mouse. Analysis of microbiota similarity between the animals with denaturing gradient gel electrophoresis revealed the development of a mouse strain-specific microbiota. Microarray-based gene expression analysis in the colonic mucosa identified 202 genes whose expression differed significantly by a factor of more than 2. Application of bioinformatics tools demonstrated that functional terms including signaling/secretion, lipid degradation/catabolism, guanine nucleotide/guanylate binding and immune response were significantly enriched in differentially expressed genes. We had a closer look at the 56 genes with expression differences of more than 4 and observed a higher expression in C57BL/10 mice of the genes coding for *Tlr1* and *Ang4* which are involved in the recognition and response to gut bacteria. A higher expression of *Pla2g2a* was detected in C3H mice. In addition, a number of interferon-inducible genes were higher expressed in C3H than in C57BL/10 mice including *Gbp1*, *Mal*, *Oasl2*, *Ifi202b*, *Rtp4, Ly6g6c*, *Ifi27l2a, Usp18*, *Ifit1*, *Ifi44*, and *Ly6g* indicating that interferons may play an essential role in microbiota regulation. However, genes coding for interferons, their receptors, factors involved in interferon expression regulation or signaling pathways were not differentially expressed between the two mouse strains. Taken together, our study confirms that the host genotype is involved in the establishment of host-specific bacterial communities in the gut. Based on expression differences after colonization with the same bacterial inoculum, we propose that *Pla2g2a* and interferon-dependent genes may contribute to this phenomenon.

## Introduction

The intestine is colonized by a complex community of bacteria. These bacteria convert indigestible food components into absorbable fermentation products and modify non-nutritive plant metabolites and drugs [1]. Deep-sequencing analyses of the gut microbiome demonstrated that a well-defined set of bacterial genes and functions is widely shared between human individuals and that a common core of bacterial species may exist [2–5]. Changes in microbiota composition or function are often observed in patients suffering from chronic disorders including inflammatory bowel diseases [6] and obesity and obesity-associated metabolic disorders [7]. It is therefore important to better understand how microbiota is shaped under physiological and pathological conditions. Diet has been considered one of the most important environmental regulators of microbiota [8]. However it can be deduced from studies in twins and less related human study subjects that the host genotype may contribute to the development of individual bacterial populations in the intestine [9,10]. The notion that the host genotype is at least in part responsible for the selection of a host-specific microbiota is supported by a study in six different genetically distinct inbred mouse strains [11].

Many genes that have been proposed to influence microbiota composition are involved in immune functions such as the pattern recognition receptors (PRRs) including Toll-like receptors (TLRs) and the nucleotide-binding oligomerization domain-containing protein 2 (NOD2). For instance, NOD2-deficient and wild-type control mice differ in microbiota composition [12,13]. Effects of TLR2-dependent mechanisms on bacterial gut colonization have been concluded from studies with a *Bacteroides fragilis* mutant strain that does not produce the capsular polysaccharide A. This strain failed to establish in the gut of germ-free mice. In contrast, the wild-type strain induced tolerance via TLR2 and successfully colonized the murine gut [14]. However, no clear effect of TLR-dependent mechanisms on gut colonization was observed when germ-free TLR2/TLR4-deficient and wild-type mice were associated with a complex bacterial inoculum from a single donor mouse [15]. Recognition of bacterial antigens by TLRs induces the expression of antimicrobial substances by Paneth cells in the small intestine [16] and Paneth cell products, namely defensins, have been shown to modulate microbiota composition [17]. Interestingly, differences in the numbers of small intestinal Paneth cells and the profiles of antimicrobial peptides produced were observed in mice which differed in their genetic background. These differences were associated with a mouse strain-specific microbiota composition [18].

Taken together, there is good indication that host-specific sensing of bacterial antigens by PRRs and the subsequent production of antimicrobial compounds is involved in the establishment of an individual microbiota. However, a large intercross study in mice suggested that complex interactions between polygenic host traits and environmental factors are responsible for microbiota individuality [19]. In order to identify candidate genes the relevance of which can later be tested in hypothesis-driven approaches, we associated germ-free inbred C57BL/10 and C3H mice with the fecal microbiota from one single conventional donor mouse. The microbiota that developed in the experimental animals was analyzed with denaturing gradient gel electrophoresis. Differences between the two mouse strains in mucosal responses towards the bacterial colonization were addressed at the gene expression level with a microarray approach and the expression of selected genes was measured with quantitative PCR.

## Materials and Methods

### Ethic statement

The protocol for the animal experiment was approved by the Animal Welfare Committee of the Ministry of Environment, Health and Consumer Protection of the Federal State of Brandenburg (Germany), State Office of Environment, Health and Consumer Protection (approval number: AZ 32-44456+1). The regulations of the German Animal Welfare Act (TierSchG, §8, Abs.1) were strictly followed.

### Animal experiment

The study was conducted in 12 week-old male C3HHeOuJ (C3H) and C57BL/10ScSn (C57BL/10) mice (12 mice per group). Animals were bred and maintained germ-free in Trexler-type isolators under highly controlled conditions (22.2 °C, 55.5% relative air humidity, 12 h light-dark cycle). Animals had free access to irradiated (25 kGy) standard chow (Altromin 1314, Altromin Spezialfutter GmbH, Lage, Germany) and autoclaved distilled drinking water. Conventional C3H and C57BL/10 (C57BL/10-MRL) mice from our breeding facilities and C57BL/10 (C57BL/10-Harlan) and C57BL/6 (C57BL/6-Harlan) mice purchased from the Harlan Laboratories (Rossdorf, Germany) were tested for mutations in the Pla2g2a-encoding gene (see below). Conventional animals were housed in individually ventilated cages under standard housing conditions.

Germ-free C3H and C57BL/10 mice were associated with the unspecified microbiota of a single C57BL/10 donor mouse. A fresh fecal sample was diluted 1:50 with sterile phosphate buffered saline (8.00 g NaCl, 0.2 g KCl, 1.44 g Na_2_HPO_4_, 0.24 g KH2PO4) and 100 µl of this suspension containing ~10^7^ bacterial cells was intragastrically applied to the experimental animals. During the experimental period, the associated mice were housed in one single isolator in polycarbonate cages on irradiated wood chips (one mouse per cage). After 13 weeks, animals were killed by cervical dislocation and colonic contents were collected for microbiota analysis. Colonic mucosa was carefully scratched, homogenized and immediately frozen in liquid nitrogen and stored at -80 °C until further processing.

### Microbiota analysis

Bacterial DNA extraction and DGGE were performed as described earlier [15]. Briefly, colonic contents and mucosa were freeze-dried and 15 mg, each, was subjected to DNA extraction with the Fast DNA SpinKit (Qbiogene, Morgan Irvine, USA). PCR was performed with the universal 16S rRNA gene-targeting primers U968-GC-f and L1401-r. DGGE was carried out with a denaturing-gradient gel electrophoresis system (C.B.S. Scientific, Del Mar, USA) using gels with a 40% to 60% denaturing gradient. The gels were subsequently silver stained [20].

### Mucosal RNA extraction and gene expression analysis

RNA was extracted from the colonic mucosa with the NucleoSpin RNA II Kit (Machery-Nagel, Duren, Germany) and shipped on dry ice to ServiceXS (Leiden, the Netherlands). At ServiceXS, the quality of the RNA was checked with the Agilent Bioanalyzer (Agilent, Santa Clara, USA). Biotin-labelled cDNA was synthesized using the Affymetrix One-Cycle Target Labeling. After testing the cDNA quality (Agilent Bioanalyzer), hybridization was performed using 12.5–20 µg of cDNA on a customized Affymetrix nugomm 1a520177 chip [21]. For unknown reasons, the RNA from one C57BL/10 mouse failed the quality tests and the mucosa obtained from this animal was excluded from all further analysis. Affymetrix protocols were strictly followed for all procedures including hybridization, washing, staining and scanning of the chips. One chip was used per experimental animal.

The relative expression of the following genes was determined by quantitative real-time PCR: *interferon-induced* guanylate-binding protein 1 (Gbp1), *Cluster of differentiation 14* (*Cd14*), *angiopoietin-4* (*Ang4*), and *phospholipase A2, group IIA* (*Pla2g2a*). *Interferon-induced guanylate-binding* protein *1*, *cluster of* differentiation *14*, and *phospholipase A2, group IIA* were selected because differences between C3H and mice with a C57 genetic background in the expression of these genes have already been published [22]. We therefore considered these genes appropriate controls. *Angiopoietin-4* was selected because it is a known Paneth cell marker [23] and we aimed at verifying its expression in the colonic mucosa of our experimental animals. *Hypoxanthine-guanine phosphoribosyltransferase* (*Hprt1*) and *ribosomal protein L13a* (*Rpl13a*) were selected as the reference genes. For quantitative real-time PCR analysis, RNA from three single mice per group and one pooled sample of the remaining mice per group were used. The RNA (0.7 µg) was transcribed with the QuantiTect Reverse Transcription Kit (Qiagen, Hilden, Germany) and quantitative real-time PCR was performed with the Applied Biosystems 7500 Fast Real-Time PCR System (Applied Biosystems, Foster City, USA) using the SYBR Green PCR kit (Qiagen). All reactions were carried out in triplicates. The primer sequences for *Gbp1* were taken from the literature [22]. The primer sequences for *Cd14* (5'- CGA ACA AGC CCG TGG AAC CT-3' and 5'-CAA GCA CAC GCT CCA TGG TC-3'), for *Ang4* (5'-TGG CCA GCT TTG GAA TCA CTG-3' and 5'-GCT TGG CAT CAT AGT GCT GAC G-3'), and for *Pla2g2a* (5'-GGC CTT TGG CTC AAT ACA GGT C-3' and 5'-ACA GTG GCA TCC ATA GAA GGC A-3') were designed with the PerlPrimer software [24]. Primer sequences for *Hprt1* and *Rpl13a* were 5'-CGT TGG GCT TAC CTC ACT GCT-3', 5'-CAT CAT CGC TAA TCA CGA CGC T-3' and 5'-GTT CGG CTG AAG CCT ACC AG-3', 5'-TTC CGT AAC CTC AAG ATC TGC T-3', respectively.

### Pla2g2a genotyping

Genomic DNA was extracted from the tail tip of three mice per group (C3H, C57BL/6, C57BL/10-MRL, C57BL/10-Harlan). The exon 3 of the Pla2g2a gene was amplified with the primers 5’-CTG GCT TTC CTT CCT GTC AGC CTG GCC-3’ and 5’-GGA AAC CAC TGG GAC ACT GAG GTA GTG-3’ [25]. The amplicons were subsequently sequenced (Eurofins MWG Operon, Ebersberg, Germany). In addition, Southern blotting using the FastDigest *Bam*HI restriction enzyme (Fermentas, St. Leon-Rot, Germany) was applied.

### Data analysis and statistics

DGGE gels were analyzed with the software GelCompar II (Applied Maths, Sint-Martens-Latem, Belgium). Differences in bacterial community structure were evaluated using the Dice similarity coefficient and bottom-up cluster analysis using the Unweighted Pair Group Method with Arithmetic mean (UPGMA). ANOVA followed by the Scheffé test was performed using SPPS 16.0 (IBM, New York, USA) in order to identify significant differences (p ≤ 0.05) between mice with the same genetic background (intra-strain differences in microbiota composition) and between the C3H and C57BL/10 mice (inter-strain differences in microbiota composition).

Mucosal affymetrix microarray gene expression data (nugomm1a520177 affymetrix arrays analyzed with nugomm1a520177mmentrezg custom cdf) was quality checked and evaluated in R (version 2.15.2) with Bioconductor (version 2.11) tools [26]. The microarray data was quality checked with affy/affyplm and normalised with Robust Multichip Average (RMA) [27]. Differential gene expression was evaluated with Linear Models For Microarray Data (limma) [28] using the false discovery rate (FDR) of 5% as the threshold for statistical significance. Gene enrichment analysis was performed with DAVID [29]. Microarray data was submitted to Gene Expression Omnibus (http://www.ncbi.nlm.nih.gov/geo/; GEO accession number: GSE45876).

The qRT-PCR data was analyzed with the 7500 software version 2.0.5 (Applied Biosystems). The relative target gene expression levels were determined with the relative standard curve method after normalization to the reference gene expression. Differences in gene expression were tested for statistical significance (p < 0.05) with the Mann-Whitney test. Prism 5.0 (Graph Pad Software, La Jolla, USA) was used for graphical data presentation and re-testing of statistical significances.

## Results

### Intestinal microbiota composition

**Figure 1:**
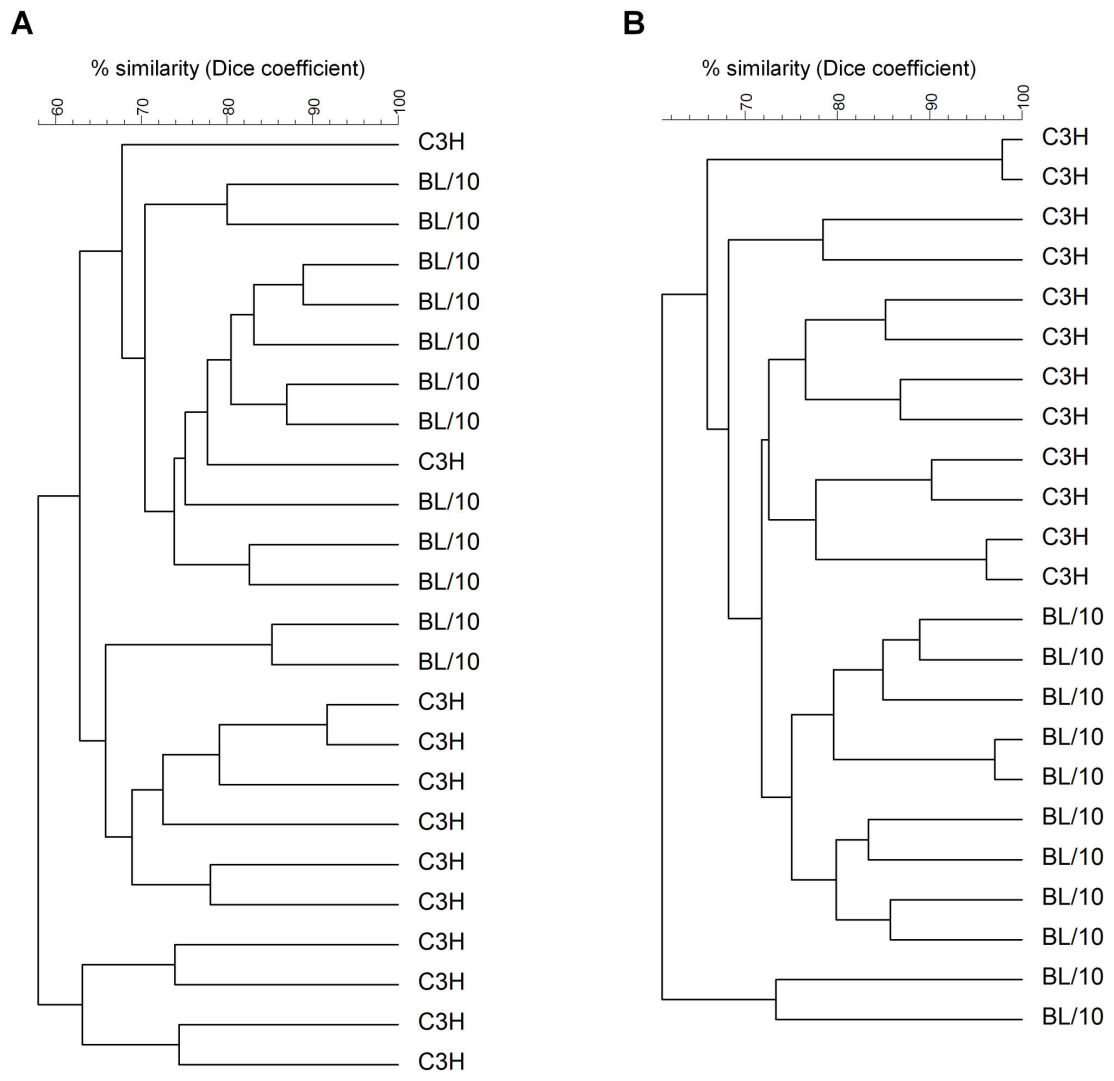
Cluster formation of C3H and C57BL/10 mice according to their luminal (A) and mucosa-associated (B) microbiota. Microbiota similarity in previously germ-free mice was calculated 13 weeks after association with the same bacterial inoculum using the Dice similarity coefficient and bottom-up cluster analysis (UPGMA) based on band patterns in DGGE gels.

Thirteen weeks after association of germ-free C3H and C57BL/10 mice with the unspecified intestinal microbiota from a single conventional C57BL/10 mouse, we analyzed the luminal and mucosa-associated microbiota composition in the colon of the recipients using a DGGE approach. The observed band patterns were used as an indicator for differences between C3H and C57BL/10 mice in microbiota composition. C3H and C57BL/10 mice grouped according to their luminal (Figure 1A) and mucosa-associated (Figure 1B) microbiota composition when cluster analysis was performed. Luminal and mucosal microbiota similarity between the C3H mice was 65.29 ± 8.93% and 72.64 ± 7.95%, respectively. The similarity between the C57BL/10 mice was 72.37 ± 8.21 for the luminal and 73.26 ± 9.78% for the mucosa-associated microbiota. The higher similarity of the luminal microbiota composition in the C57BL/10 mice was statistically significant (p < 0.001). When the band patterns of the C57BL/10 was compared with the patterns of the C3H mice, the similarity in microbiota composition was 61.40 ± 8.86% in the lumen and 67.70 ± 7.55% at the colonic wall. The statistical analysis revealed that the intra-strain similarity in the lumen (p = 0.011) and at the mucosal wall (p < 0.001) was significantly higher than the inter-strain similarity. A similarity matrix showing the microbiota similarity in per-cent between the experimental animals is presented in Table S1.

#### Differences in mucosal gene expression

We compared the expression of ~16.000 genes in the colonic mucosa of previously germ-free C3H and C57BL/10 mice 13 weeks after association with intestinal bacteria. The expression of 210 genes significantly (FDR ≤ 0.05) differed by a factor of ≥ 2 (Table S2). Twenty-six of these genes were > 4-fold higher expressed in C57BL/10 (Table 1) and 30 genes were > 4-fold higher expressed in C3H mice (Table 2). DAVID functional gene enrichment analysis was performed on all 210 differentially expressed genes. Significantly enriched functional terms identified were signaling/secretion, lipid degradation/catabolism, guanine nucleotide/guanylate binding and immune response. These terms included 66 genes associated with signaling/secretion (Table S3), 7 with peptidase inhibition (Table S4), 6 with guanine binding (Table S5), 18 with response to bacteria/defense (Table S6), 7 with hormone activity (Table S7) and 14 lipoprotein-associated genes (Table S8).

**Table 1:** Genes > 4-fold higher expressed in C57BL/10 than in C3H mice (FDR ≤ 0.05).

**Gene symbol**	**Gene name**	**Fold change**
Ang4	angiogenin, ribonuclease A family, member 4	33.06
Lin7c	lin-7 homolog C (C. elegans)	32.64
Nxpe4	neurexophilin and PC-esterase domain family, member 4	30.51
Pla2g4c	phospholipase A2, group IVC (cytosolic, calcium-independent)	23.41
Pnliprp2	pancreatic lipase-related protein 2	15.17
Spna1	spectrin alpha 1	13.64
Rpgrip1	retinitis pigmentosa GTPase regulator interacting protein 1	13.09
Pdxdc1	pyridoxal-dependent decarboxylase domain containing 1	12.12
Fam199x	family with sequence similarity 199, X-linked	11.43
Pcdh17	protocadherin 17	11.37
B4galnt2	beta-1,4-N-acetyl-galactosaminyl transferase 2	9.31
Cbr3	carbonyl reductase 3	8.45
Trim30d	tripartite motif-containing 30D	8.14
Mep1a	meprin 1 alpha	7.77
Trim34a	tripartite motif-containing 34A	7.19
Ceacam12	carcinoembryonic antigen-related cell adhesion molecule 12	6.10
Gsdmc4	gasdermin C4	5.76
P2ry6	pyrimidinergic receptor P2Y, G-protein coupled, 6	5.27
Msi2	Musashi homolog 2 (Drosophila)	5.02
1810030J14Rik	RIKEN cDNA 1810030J14 gene	4.66
Myl7	myosin, light polypeptide 7, regulatory	4.40
Tlr1	toll-like receptor 1	4.34
Itln1	intelectin 1 (galactofuranose binding)	4.27
Gsdmc2	gasdermin C2	4.21
2210407C18Rik	RIKEN cDNA 2210407C18 gene	4.21
Fam73a	family with sequence similarity 73, member A	4.10

**Table 2:** Genes > 4-fold higher expressed in C3H than in C57BL/10 mice (FDR ≤ 0.05).

**Gene symbol**	**Gene name**	**Fold change**
Pla2g2a	phospholipase A2, group IIA (platelets, synovial fluid)	70.80
Nxpe5	neurexophilin and PC-esterase domain family, member 5	33.03
Slpi	secretory leukocyte peptidase inhibitor	28.44
Qpct	glutaminyl-peptide cyclotransferase (glutaminyl cyclase)	18.57
Gbp1	guanylate binding protein 1	18.38
Plscr2	phospholipid scramblase 2	16.62
Lpo	lactoperoxidase	11.25
Ppy	pancreatic polypeptide	10.39
Apoc2	apolipoprotein C-II	8.96
Abhd1	abhydrolase domain containing 1	8.87
Mal	myelin and lymphocyte protein, T cell differentiation protein	7.13
Oasl2	2'-5' oligoadenylate synthetase-like 2	6.67
Ifi202b	interferon activated gene 202B	6.51
Afm	afamin	6.47
Hddc3	HD domain containing 3	6.46
Rtp4	receptor transporter protein 4	5.64
BC064078	cDNA sequence BC064078	5.64
Ly6g6c	lymphocyte antigen 6 complex, locus G6C	5.46
Tff2	trefoil factor 2 (spasmolytic protein 1)	5.45
Amn	amnionless	5.44
Ifi27l2a	interferon, alpha-inducible protein 27 like 2A	5.23
Usp18	ubiquitin specific peptidase 18	5.00
Tmem87a	transmembrane protein 87A	4.69
Ifit1	interferon-induced protein with tetratricopeptide repeats 1	4.56
2610305D13Rik	RIKEN cDNA 2610305D13 gene	4.55
Ifi44	interferon-induced protein 44	4.34
Cd14	CD14 antigen	4.17
2210010C17Rik	RIKEN cDNA 2210010C17 gene	4.13
Ly6g	lymphocyte antigen 6 complex, locus G	4.11
Eno3	enolase 3, beta muscle	4.05

We had a closer look at the functions of genes with fold changes of more than 4. Many of these genes are involved in the synthesis of antibacterial factors and in immune functions Genes with (possible) antibacterial functions include the *phospholipase A2, group IIA*, *secretory leukocyte peptidase inhibitor*, and *lactoperoxidase* all of which were higher expressed in C3H mice. In contrast, the *angiogenin, ribonuclease* *A* *family* *,* member *4* was higher expressed in the BL/10 mice. The LPS receptor *cluster of* differentiation *14* was higher expressed in C3H mice and the *toll-like* receptor *1* was higher expressed in the BL/10 animals. Interferon-inducible genes were higher expressed in C3H than in C57BL/10 mice. These genes included *guanylate nucleotide binding* protein *1*, *myelin and lymphocyte protein*, *2*'*-5*' *oligoadenylate synthetase-like 2*, *interferon activated gene 202B*, *receptor transporter* protein *4*, *lymphocyte antigen 6 complex, locus G6C*, *interferon alpha-inducible* protein *27 like 2A*, *ubiquitin-specific peptidase 18*, *interferon-induced protein with tetratricopeptide repeats 1*, *interferon-induced* protein *44*, and *lymphocyte antigen 6 complex, locus G*.

**Figure 2:**
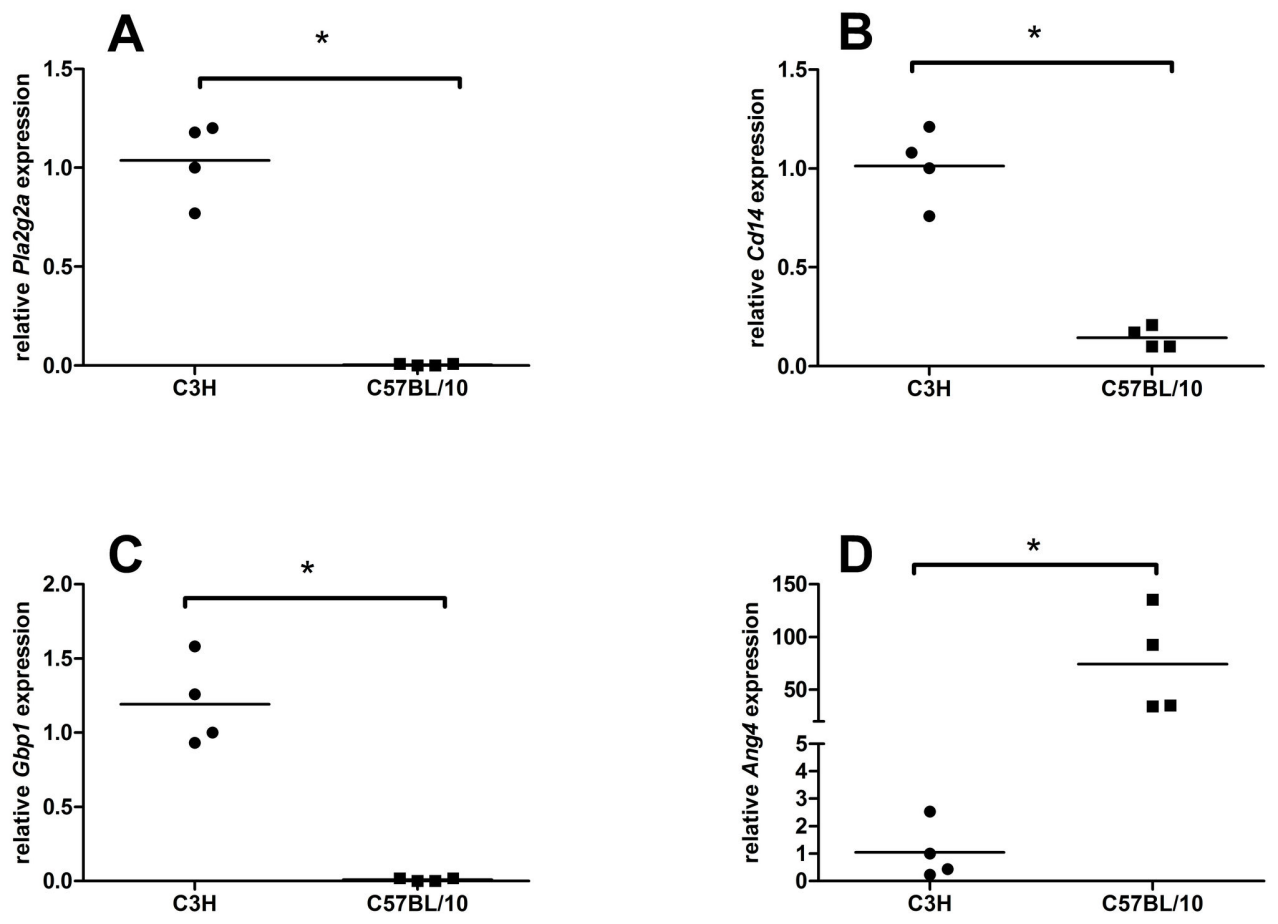
Quantitative real-time results for the expression of *Pla2g2a* (A), *CD14* (B) *Gbp1* (C) *and Ang4* (2D) in the colonic mucosa of previously germ-free C3H and C57BL/10 mice 13 weeks after association with the same bacterial inoculum. RNA from three single mice per group and pooled RNA from the remaining C3H and C57BL/10 mice, respectively were used. *Hypoxanthine-guanine phosphoribosyltransferase* (*Hprt1*) and *ribosomal protein L13a* (*Rpl13a*) expression were selected for data normalization and tested for tested for statistical significance (* < 0.05) with the Mann-Whitney test.

The expression at the mRNA level of four selected genes was measured with qRT-PCR to confirm the microarray gene data. The relative expression of *phospholipase A2, group IIA* (*Pla2g2a*), *cluster of* differentiation *14* (*CD14*) and *guanylate nucleotide binding* protein *1* (*Gbp1*) was higher in the colonic mucosa of the C3H than in C57BL/10 mice. In contrast, the expression of *Ang4* was significantly higher in C57BL/10 than in C3H mice (Figure 2). These findings are in line with the results from the microarray experiment.

### Testing for frameshift mutations in the Pla2g2a-encoding gene

C57BL/10 and C57BL/6 mice share a considerable proportion of their genetic background. Since the Pla2g2a gene is naturally disrupted in C57BL/6 mice by a frameshift mutation in exon 3, we tested whether this was also the case in our experimental C57BL/10. In addition, we sequenced the gene in the experimental C3H mice and in conventional C57BL/10 mice purchased from the Harlan laboratories. C57BL/6 mice of the same origin were used as a reference. We did not detect the frameshift mutation in our experimental C57BL/10 mice or in any C3H mouse when the exon 3 was sequenced. This result was confirmed by Southern blotting which clearly demonstrated that both (sub-) strains used are homozygous for the wild-type Pla2g2a genotype. In contrast, C57BL/6 mice and C57BL/10 purchased from Harlan laboratories were homozygous for the defective Pla2g2a gene.

## Discussion

Interactions between intestinal bacteria and the host at the mucosal interface influence host health and disease. For instance, excessive host responses towards commensal gut bacteria are associated with the onset and perpetuation of chronic gut inflammation [6]. On the other hand, host factors may influence gut bacteria since differences in the recognition of and in responses towards intestinal bacteria are implicated in the selection of individual intestinal microbiota [30]. However, the exact nature and the mechanisms of such interactions are poorly understood. To provide deeper insight into host-specific responses to commensal gut bacteria, we associated germ-free C3H and C57BL/10 mice with the very same bacterial inoculum. Upon association, the mice developed a strain-specific intestinal microbiota composition which is in line with our previous observations in these mouse strains and with findings by others [15,31–33].

To identify genotype-specific responses towards bacterial colonization of the intestine, we analyzed with a microarray approach the mucosal gene expression in our experimental animals. This technique has been previously applied by others to describe differences in intestinal gene expression between germ-free and conventional C3H mice revealing that more than 50% of the differentially regulated genes were higher expressed in the conventional mice. The majority of these genes grouped in Gene Ontology biological processes involved in water transport across the gut wall and immune responses. Since mainly immunoglobulin-associated genes were identified in the “defense/immunity protein activity molecular function cluster”, it can be assumed that presence of microbiota influenced adaptive immune responses in this model [34]. The category “immune responses” was also enriched in differentially expressed genes when mucosal gene expression in our experimental animals was evaluated with bioinformatics approaches. We had a closer look at single genes whose expression differed by a factor of greater than 4. Amongst these genes, we found higher expression in C57BL/10 than in C3H mice of toll-like receptor 1 (TLR1, 4-fold) and *angiogenin 4* (*Ang4*, 33-fold). Direct effects of these two genes on colonic microbiota composition have so far not been demonstrated. However, differences in *Ang4* expression have been reported between germ-free mice and mice monoassociated with *Bacteroides* *thetaiotaomicron* [35]. The antibacterial peptide Ang4 is very effective against Gram positive bacteria including *Enterococcus faecalis* and *Listeria monocytogenes*. Gram negative bacteria such as *Bacteroides* *thetaiotaomicron* and *Escherichia coli* are less sensitive. Interestingly, intestinal *Ang4* mRNA expression is induced by complex microbiota and by *B* *. thetaiotaomicron* alone in previously germ-free mice [23].

In contrast to *Ang4*, *phospholipase A2, group IIA* (*Pla2g2a*), *guanylate nucleotide binding* protein *1* (*Gbp1*), and *cluster of* differentiation *14* (*Cd14*) were higher expressed in C3H than in C57BL/10 mice. The same observation has been reported in C3H and C57BL/6 mice and was associated with a strain-specific susceptibility to microbiota-driven colitis: interleukin 10-deficient mice with a C3H background are susceptible whereas the C57BL/6 background renders the mice colitis resistant [22]. The latter strain shares a considerable proportion of its genetic background with the C57BL/10 mice that were used in our experiment because C57BL/6 and C57BL/10 are sub-strains of C57BL origin. For *Cd14*, which is involved in the detection of lipopolysaccharides by TLR4, it was later demonstrated that a higher expression by gut epithelial cells is responsible for a lower colitis susceptibility in IL-10^-/-^ mice with a C3H than with a C57BL/6 background [36]. Whether or not differences in intestinal Cd14 expression are also involved in the selection of resident gut bacteria remains to be clarified.

The high genetic homogeneity that can be assumed for C57BL/6 and C57BL/10 mice suggests that our experimental mice were affected by a known frameshift mutation in exon 3 of the *Pla2g2a* gene in C57BL/6 mice which results in a defective gene product [25]. This would have been the most plausible explanation for a more than 70-fold higher expression of this gene in C3H mice. However, gene sequence and Southern blot analyses revealed that this mutation was absent in our experimental animals. Differences in intestinal Pla2g2a activities have been shown for different mouse strains with the wild-type alleles. It has been proposed in one study that nucleotide substitutions and resulting amino acid changes may influence the substrate binding properties of the enzyme [37]. In conjunction with the fact that *Pla2g2a* gene expression is equal in germ-free and conventional mice [23], these findings suggest that this gene is a good candidate for genetically fixed host factors that may influence microbiota composition. In fact, Pla2g2a exerts strong antibacterial effects against Gram positive and to a lesser extend against Gram negative bacteria and there is evidence that it plays an important role in the intestinal defense against pathogenic bacteria [38].

The expression in human bronchioepithelial and nasal epithelial cells of type IIA phospholipase A2 is up-regulated by IFN-gamma [39] indicating that interferons may regulate the expression of genes with possible functions in host-microbe interactions. This notion is supported by the fact that many interferon (IFN)-inducible genes were > 4-fold higher expressed in C3H than in C57BL/10 mice. In addition to the aforementioned *Gbp1*, these genes included *Mal*, *Oasl2*, *Ifi202b*, *Rtp4, Ly6g6c*, *Ifi27l2a, Usp18*, *Ifit1*, *Ifi44*, and *Ly6g*. Interestingly, the interferon regulatory factor 9-encoding gene (*IRF9*) was 2-fold higher expressed in C3H mice. It may therefore well be that IFN-dependent epithelial responses towards gut bacteria differ in a host-specific manner. However, whether individual IFN-dependent immune responses are the cause or the consequence of differences in intestinal microbiota composition remains elusive. On the one hand mouse strain specific *Gbp1* expression has been demonstrated. Among the 46 inbred strains tested, mice with a C3H but not with a C57BL background displayed IFN-inducible GBP-1 synthesis [40]. The importance of genes that are regulated by interferon-alpha or interferon-beta has been concluded from experiments with IRF9-deficient mice. These mice with an impaired IFN-alpha and IFN-beta signaling show higher temporal variations in and lower individuality of intestinal microbiota composition than wild-type control mice and STAT1-deficient mice with defective IFN-gamma signaling [41]. On the other hand intestinal bacteria influence the expression of IFN-inducible genes. For instance, differential expression profiles of IFN-responsive genes in response to monoassociation with *Bacteroides* *thetaiotaomicron* and *Bifidobacterium* *longum* have been observed in mice. Based on gene interaction network analysis it has been concluded that host responses to *B* *. thetaiotaomicron* are centered on tumor necrosis factor alpha triggered gene expression but that the gene expression network associated with *Bifidobacterium* *longum* is centered on INF-gamma responsive genes [42]. Taken together, there is good indication that interferons are important players in host-bacteria interactions. However, we did not observe differences between C3H and C57BL/10 mice in the expression of genes coding for interferons, their receptors or for molecules involved in interferon signaling.

Differentially expressed genes with functions in the immune system included *SLPI* encoding the secretory leucocyte protease inhibitor (28-fold higher expressed in C3H) and *Mep1a* coding for meprin 1 α (8-fold higher expressed in C57BL/10). *SLPI* expression is up-regulated by bacterial antigens and under inflammatory conditions. The protein protects the tissue from immune cell-derived proteases and is implicated in the priming of innate immunity and tissue repair. It also acts as an antibacterial protein and is active against both Gram positive and Gram negative bacteria [43]. Meprins have been implicated in dysregulated immune responses towards gut bacteria in chronic gut inflammation because the expression of meprins is down-regulated in patients with ileal Crohn’s disease. Pre-treatment with meprin α and meprin β decreased the ability of *E. coli* to bind to host receptors and to induce the expression of pro-inflammatory IL-8 [44]. These findings suggest that SLPI and meprin 1 α may act as microbiota regulators.

The intestinal mucus layer in the colon consists of a dense inner bacteria-free and a loose outer layer that is colonized by commensal bacteria. The mucins that form this layer are mainly composed of the densely *O*-glycosylated mucus protein MUC2. The glycans may serve as substrates and, in addition as adhesion sites for gut bacteria. Therefore, different mucin glycosylation pattern may be involved in the selection of a host specific microbiota [45]. Interestingly, a recent study clearly demonstrated that intestinal expression of *β-*1,4-*N-acetylgalactosaminyltransferase 2* (*B4galnt2*) which may contribute to mucin decoration with *N*‑acetylgalactosamine influences gut microbiota in mice [46]. Using a 16S rRNA gene sequencing approach, the authors identified numerous bacterial taxa or operational taxonomic units that were influenced by *B4galnt2* expression. Since closely related bacterial species appeared to replace each other in *B4galnt2* ^*-/-*^ *and B4galnt2* ^*+/+*^ mice, respectively, it is difficult to interpret whether *B4galnt2*-induced changes influence microbiota function. Interestingly, *Helicobacter* spp. were rarely detected in the intestine of *B4galnt2* ^*-/-*^ mice indicating that adhesion of the pathogen and possibly by other adherent bacteria to *N*-acetogalactosamine residues in the intestine is impaired in these animals.

Genes with functions in plasma membrane structure or functions (*Lin7c*, *Plscr2*, *AMN*) and cell adhesion and cell signaling (*Pcdh17, P2ry6, Ceacam12*) were also differentially regulated in the experimental mice but their role in intestinal host-microbe interactions was not obvious from literature research.

In summary, differences between inbred mouse strains in microbiota composition suggest that genetic host factors are involved in the selection of host-specific bacteria. From our findings we conclude that *Pla2ga2* and interferon-responsive genes are good candidates for the identification of host factors that play a role in host-microbe interactions. The specific role of these candidate genes may be addressed in hypothesis-driven experiments taking advantage of gnotobiotic knockout mouse models. In addition, it may be possible in such studies to clarify whether differential expression of selected genes is cause or consequence of differences in intestinal microbiota composition.

## Supporting Information

Table S1 **Similarity of the intestinal microbiota in the colonic lumen **(**A**) **and at the clonic mucosa **(**B**).
(PDF)

Table S2 **Genes differentially expressed (fold change >2) between C57BL/10 and C3H mice.**
(PDF)

Table S3 **DAVID functional gene list: signaling/secretion.**
(PDF)

Table S4 **DAVID functional gene list: peptidase inhibition.**
(PDF)

Table S5 **DAVID functional gene list: guanine binding.**
(PDF)

Table S6 **DAVID functional gene list: response to bacteria/defense.**
(PDF)

Table S7 **DAVID functional gene list: hormone activity.**
(PDF)

Table S8 **DAVID functional gene list: lipoprotein-associated.**
(PDF)
