# Supplementary material for: Differences in Mucosal Gene Expression in the Colon of Two Inbred Mouse Strains after Colonization with Commensal Gut Bacteria
Source: PLoS One. 2013 Aug 9;8(8):e72317. doi: 10.1371/journal.pone.0072317 (PMC3739790; doi:10.1371/journal.pone.0072317)
Supplement: Table S1 — Similarity of the intestinal microbiota in the colonic lumen (A) and at the clonic mucosa (B). (PDF) [file pone.0072317.s001.pdf]

Table S1: Similarity of the intestinal microbiota in the colonic lumen (A) and at the clonic mucosa (B)

A: Similarity of the microbiota in the colonic lumen of the experimental animals (%)

	C3H_1	C3H_2	C3H_3	C3H_4	C3H_5	C3H_6	C3H_7	C3H_8	C3H_9	C3H_10	C3H_11	C3H_12	B10_1	B10_2	B10_3	B10_4	B10_5	B10_6	B10_7	B10_8	B10_9	B10_10	B10_11	B10_12
**C3H_1**	100.00																							
**C3H_2**	62.5	100.00																						
**C3H_3**	66.67	63.64	100.00																					
**C3H_4**	69.23	52.39	91.67	100.00																				
**C3H_5**	75.47	65.12	81.63	76.60	100.00																			
**C3H_6**	60.00	60.00	78.26	68.19	71.11	100.00																		
**C3H_7**	55.32	75.68	69.77	58.54	76.19	66.67	100.00																	
**C3H_8**	69.23	76.19	79.17	65.22	76.60	59.09	78.05	100.00																
**C3H_9**	61.54	57.15	62.5	60.87	63.83	68.19	53.66	60.87	100.00															
**C3H_10**	57.69	57.15	66.67	52.17	63.83	50.00	48.78	65.22	73.91	100.00														
**C3H_11**	50.00	52.63	72.73	66.67	55.81	70.00	54.06	61.91	66.67	66.67	100.00													
**C3H_12**	64.15	60.47	73.47	51.07	66.67	62.23	71.43	76.6	59.58	59.58	74.42	100.00												
**B10_1**	61.54	57.15	58.33	65.22	51.07	50.00	58.54	60.87	43.48	43.48	42.86	63.83	100.00											
**B10_2**	58.83	68.29	55.32	53.33	47.83	46.51	65.00	57.78	44.45	44.45	43.91	65.22	80.01	100.00										
**B10_3**	73.08	76.19	54.17	52.17	68.09	63.64	78.05	65.22	52.17	52.17	52.39	72.35	69.57	75.56	100.00									
**B10_4**	74.51	82.93	63.83	53.33	60.87	60.47	75.00	66.67	57.78	62.23	53.66	69.57	62.23	72.73	88.89	100.00								
**B10_5**	66.67	72.35	60.38	58.83	65.39	61.23	69.57	62.75	62.75	66.67	51.07	65.39	70.59	72.00	86.28	80.01	100.00							
**B10_6**	73.08	80.95	62.50	60.87	63.83	59.09	78.05	78.26	52.17	56.53	42.86	63.83	69.57	84.45	78.26	84.45	74.51	100.00						
**B10_7**	76.93	76.19	62.50	56.53	72.35	72.73	68.29	69.57	60.87	60.87	47.62	63.83	69.57	75.56	82.61	84.45	78.43	86.96	100.00					
**B10_8**	64.15	69.77	61.23	46.81	58.33	62.23	71.43	55.32	51.07	55.32	46.51	62.5	63.83	65.22	80.85	82.61	76.93	68.09	72.35	100.00				
**B10_9**	72.73	71.11	62.75	57.15	60.00	55.32	68.19	65.31	48.98	53.07	53.33	72.00	69.39	70.83	81.63	79.17	81.49	69.39	69.39	72.00	100.00			
**B10_10**	61.23	66.67	62.23	55.81	54.55	48.78	73.69	65.12	55.81	55.81	41.03	68.19	69.77	80.95	79.07	76.19	75.00	74.42	69.77	68.19	82.61	100.00		
**B10_11**	61.29	61.54	68.97	60.72	70.18	66.67	66.67	67.86	60.72	53.57	53.85	66.67	57.15	69.09	67.86	61.82	72.13	71.43	67.86	63.16	64.41	56.61	100.00	
**B10_12**	66.67	68.09	64.15	62.75	73.08	65.31	60.87	62.75	62.75	54.91	42.55	57.69	54.91	64.00	62.75	64.00	67.86	66.67	70.59	61.54	62.97	58.33	85.25	100.00

B: Similarity of the microbiota at the colonic mucosa of the experimental animals (%)

	C3H_1	C3H_2	C3H_3	C3H_4	C3H_5	C3H_6	C3H_7	C3H_8	C3H_9	C3H_10	C3H_11	C3H_12	B10_1	B10_2	B10_3	B10_4	B10_5	B10_6	B10_7	B10_8	B10_9	B10_10	B10_11
**C3H_1**	100.00																						
**C3H_2**	97.87	100.00																					
**C3H_3**	66.67	68.01	100.00																				
**C3H_4**	62.5	59.58	78.43	100.00																			
**C3H_5**	75.47	69.23	82.15	79.25	100.00																		
**C3H_6**	77.55	70.83	69.23	69.39	85.19	100.00																	
**C3H_7**	74.51	64.00	70.37	66.67	82.15	73.08	100.00																
**C3H_8**	72.00	69.39	60.38	64.00	76.37	74.51	86.79	100.00															
**C3H_9**	65.31	66.67	61.54	69.39	77.78	68.01	80.77	74.51	100.00														
**C3H_10**	72.00	61.23	64.15	68.01	76.37	70.59	75.47	76.93	90.2	100.00													
**C3H_11**	74.51	76.00	70.37	62.75	71.43	69.23	77.78	71.7	73.08	83.02	100.00												
**C3H_12**	73.47	75.00	69.23	61.23	66.67	64.00	73.08	66.67	80.01	74.51	96.15	100.00											
**B10_1**	61.54	58.83	76.37	69.23	80.71	75.47	76.37	70.37	79.25	81.49	76.37	71.70	100.00										
**B10_2**	64.00	57.15	75.47	68.01	69.09	70.59	75.47	65.39	70.59	73.08	71.70	66.67	88.89	100.00									
**B10_3**	68.01	57.15	71.70	60.00	72.73	66.67	75.47	69.23	70.59	76.93	83.02	78.43	85.19	84.62	100.00								
**B10_4**	67.80	65.52	77.42	64.41	81.25	73.33	77.42	68.85	73.33	68.85	64.52	66.67	85.72	81.97	75.41	100.00							
**B10_5**	66.67	60.72	76.67	66.67	80.65	72.41	76.67	67.8	75.87	71.19	60.60	68.97	85.25	77.97	71.19	97.06	100.00						
**B10_6**	60.87	53.33	69.39	73.91	74.51	72.35	73.47	66.67	63.83	66.67	65.31	59.58	76.00	75.00	75.00	70.18	69.09	100.00					
**B10_7**	60.00	61.23	71.10	60.00	80.01	70.59	75.47	73.08	70.59	76.93	71.7	66.67	85.19	76.93	80.77	75.41	77.97	83.33	100.00				
**B10_8**	66.67	63.64	66.67	62.23	68.01	65.22	75.00	72.35	78.26	80.85	75.00	73.91	81.63	76.60	80.85	67.86	70.37	79.07	85.11	100.00			
**B10_9**	62.23	54.55	62.50	57.78	68.01	56.53	70.83	63.83	65.22	76.60	70.83	65.22	81.63	72.35	76.60	64.29	66.67	74.42	80.85	85.72	100.00		
**B10_10**	55.56	51.43	61.54	55.56	53.66	54.06	56.41	52.63	59.46	52.63	56.41	59.46	60.00	63.16	57.90	51.07	53.33	58.83	57.90	66.67	60.61	100.00	
**B10_11**	57.15	53.66	71.11	66.67	68.09	60.47	71.11	63.64	60.47	59.09	62.23	65.12	65.22	68.19	68.19	64.15	66.67	75.00	63.64	71.80	61.54	73.33	100.00
