# Supplementary material for: Differences in Mucosal Gene Expression in the Colon of Two Inbred Mouse Strains after Colonization with Commensal Gut Bacteria
Source: PLoS One. 2013 Aug 9;8(8):e72317. doi: 10.1371/journal.pone.0072317 (PMC3739790; doi:10.1371/journal.pone.0072317)
Supplement: Table S2 — Genes differentially expressed (fold change >2) between C57BL/10 and C3H mice. (PDF) [file pone.0072317.s002.pdf]

Table S2: Genes differentially expressed (fold change >2) between C57BL/10 and C3H mice

Gene Symbol	Gene Name	Fold change	FDR	Higher expressed in
Pla2g2a	phospholipase A2, group IIA (platelets, synovial fluid)	70,80	1,62E-17	C3H
Ang4	angiogenin, ribonuclease A family, member 4	33,06	2,23E-07	C57BL/10
Nxpe5	neurexophilin and PC-esterase domain family, member 5	33,03	3,84E-16	C3H
Lin7c	lin-7 homolog C (C. elegans)	32,64	3,84E-16	C57BL/10
Nxpe4	neurexophilin and PC-esterase domain family, member 4	30,51	1,43E-11	C57BL/10
Slpi	secretory leukocyte peptidase inhibitor	28,44	5,23E-14	C3H
Pla2g4c	phospholipase A2, group IVC (cytosolic, calcium-independent)	23,41	8,76E-08	C57BL/10
Qpct	glutaminyl-peptide cyclotransferase (glutaminyl cyclase)	18,57	1,04E-18	C3H
Gbp1	guanylate binding protein 1	18,38	4,77E-16	C3H
Plscr2	phospholipid scramblase 2	16,62	3,11E-14	C3H
Pnliprp2	pancreatic lipase-related protein 2	15,17	2,19E-08	C57BL/10
Spna1	spectrin alpha 1	13,64	2,93E-08	C57BL/10
Rpgrip1	retinitis pigmentosa GTPase regulator interacting protein 1	13,09	1,09E-15	C57BL/10
Pdxdc1	pyridoxal-dependent decarboxylase domain containing 1	12,12	1,04E-18	C57BL/10
Fam199x	family with sequence similarity 199, X-linked	11,43	8,40E-18	C57BL/10
Pcdh17	protocadherin 17	11,37	4,98E-12	C57BL/10
Lpo	lactoperoxidase	11,25	1,04E-11	C3H
Ppy	pancreatic polypeptide	10,39	8,83E-11	C3H
B4galnt2	beta-1,4-N-acetyl-galactosaminyl transferase 2	9,31	2,85E-05	C57BL/10
Apoc2	apolipoprotein C-II	8,96	1,97E-11	C3H
Abhd1	abhydrolase domain containing 1	8,87	1,71E-14	C3H
Cbr3	carbonyl reductase 3	8,45	4,98E-12	C57BL/10
Trim30d	tripartite motif-containing 30D	8,14	1,09E-05	C57BL/10
Mep1a	meprin 1 alpha	7,77	8,16E-13	C57BL/10
Trim34a	tripartite motif-containing 34A	7,19	5,52E-12	C57BL/10
Mal	myelin and lymphocyte protein, T cell differentiation protein	7,13	6,05E-07	C3H
Oasl2	2'-5' oligoadenylate synthetase-like 2	6,67	6,68E-09	C3H
Ifi202b	interferon activated gene 202B	6,51	3,13E-06	C3H
Afm	afamin	6,47	2,07E-15	C3H
Hddc3	HD domain containing 3	6,46	4,25E-16	C3H
Ceacam12	carcinoembryonic antigen-related cell adhesion molecule 12	6,10	2,15E-04	C57BL/10
Gsdmc4	gasdermin C4	5,76	7,44E-12	C57BL/10
Rtp4	receptor transporter protein 4	5,64	1,40E-10	C3H
BC064078	cDNA sequence BC064078	5,64	1,42E-12	C3H

Table S1 (continued): Genes differentially expressed (fold change >2) between C57BL/10 and C3H mice

Gene Symbol	Gene Name	Fold change	FDR	Higher expressed in
Ly6g6c	lymphocyte antigen 6 complex, locus G6C	5,46	2,71E-07	C3H
Tff2	trefoil factor 2 (spasmolytic protein 1)	5,45	1,24E-04	C3H
Amn	amnionless	5,44	4,76E-09	C3H
P2ry6	pyrimidinergic receptor P2Y, G-protein coupled, 6	5,27	1,54E-12	C57BL/10
Ifi27l2a	interferon, alpha-inducible protein 27 like 2A	5,23	1,26E-05	C3H
Msi2	Musashi homolog 2 (Drosophila)	5,02	5,40E-11	C57BL/10
Usp18	ubiquitin specific peptidase 18	5,00	2,28E-07	C3H
Tmem87a	transmembrane protein 87A	4,69	4,64E-15	C3H
1810030J14Rik	RIKEN cDNA 1810030J14 gene	4,66	7,66E-04	C57BL/10
Ifit1	interferon-induced protein with tetratricopeptide repeats 1	4,56	2,01E-04	C3H
2610305D13Rik	RIKEN cDNA 2610305D13 gene	4,55	2,48E-13	C3H
Myl7	myosin, light polypeptide 7, regulatory	4,40	5,84E-09	C57BL/10
Tlr1	toll-like receptor 1	4,34	8,16E-13	C57BL/10
Ifi44	interferon-induced protein 44	4,34	1,05E-04	C3H
Itln1	intelectin 1 (galactofuranose binding)	4,27	2,63E-02	C57BL/10
2210407C18Rik	RIKEN cDNA 2210407C18 gene	4,21	4,00E-04	C57BL/10
Gsdmc2	gasdermin C2	4,21	7,61E-11	C57BL/10
Cd14	CD14 antigen	4,17	1,11E-10	C3H
2210010C17Rik	RIKEN cDNA 2210010C17 gene	4,13	1,71E-14	C3H
Ly6g	lymphocyte antigen 6 complex, locus G	4,11	4,30E-03	C3H
Fam73a	family with sequence similarity 73, member A	4,10	1,43E-12	C57BL/10
Eno3	enolase 3, beta muscle	4,05	1,44E-03	C3H
Tns4	tensin 4	3,95	2,68E-08	C57BL/10
Cnot7	CCR4-NOT transcription complex, subunit 7	3,83	9,00E-12	C3H
Clps	colipase, pancreatic	3,79	2,07E-06	C57BL/10
S100g	S100 calcium binding protein G	3,67	1,54E-02	C57BL/10
Cd74	CD74 antigen (invariant polypeptide of major histocompatibility complex, class II antigen-associated)	3,61	2,27E-07	C3H
Gsdmcl-ps	gasdermin C-like, pseudogene	3,56	3,19E-07	C57BL/10
Ifit2	interferon-induced protein with tetratricopeptide repeats 2	3,53	2,08E-07	C3H
Dixdc1	DIX domain containing 1	3,49	9,96E-05	C57BL/10
Col8a1	collagen, type VIII, alpha 1	3,46	5,90E-04	C3H
Wars	tryptophanyl-tRNA synthetase	3,45	6,06E-14	C57BL/10
Insl5	insulin-like 5	3,44	1,74E-03	C3H
Herc6	hect domain and RLD 6	3,41	2,31E-06	C3H

Table S1 (continued): Genes differentially expressed (fold change >2) between C57BL/10 and C3H mice

Gene Symbol	Gene Name	Fold change	FDR	Higher expressed in
D330028D13Rik	RIKEN cDNA D330028D13 gene	3,40	1,04E-06	C57BL/10
Isoc2b	isochorismatase domain containing 2b	3,36	1,95E-14	C57BL/10
H2-Bl	histocompatibility 2, blastocyst	3,20	8,76E-08	C3H
Zfp462	zinc finger protein 462	3,19	2,66E-06	C57BL/10
Mtmr7	myotubularin related protein 7	3,17	7,03E-12	C57BL/10
Zfp939	zinc finger protein 939	3,15	5,70E-10	C57BL/10
Ccdc109b	coiled-coil domain containing 109B	3,13	6,19E-09	C57BL/10
Defb37	defensin beta 37	3,13	1,86E-03	C57BL/10
Rps3	ribosomal protein S3	3,10	2,71E-10	C57BL/10
Hoxb6	homeobox B6	3,10	3,74E-02	C57BL/10
Alpi	alkaline phosphatase, intestinal	3,09	6,69E-03	C3H
1700011H14Rik	RIKEN cDNA 1700011H14 gene	3,08	2,85E-04	C57BL/10
Airn	antisense Igf2r RNA	2,98	7,52E-07	C57BL/10
Crim1	cysteine rich transmembrane BMP regulator 1 (chordin like)	2,98	6,90E-11	C57BL/10
Ermap	erythroblast membrane-associated protein	2,97	3,57E-03	C57BL/10
Comt	catechol-O-methyltransferase	2,95	7,44E-12	C3H
Fkbp1b	FK506 binding protein 1b	2,94	2,00E-06	C3H
4833420G17Rik	RIKEN cDNA 4833420G17 gene	2,93	5,84E-09	C57BL/10
Apol6	apolipoprotein L 6	2,90	2,19E-06	C3H
Lyz2	lysozyme 2	2,87	1,10E-05	C3H
Dcaf17	DDB1 and CUL4 associated factor 17	2,86	2,94E-09	C57BL/10
Tor3a	torsin family 3, member A	2,86	3,78E-10	C3H
Ptprf	protein tyrosine phosphatase, receptor type, F	2,85	1,20E-10	C3H
Ddx60	DEAD (Asp-Glu-Ala-Asp) box polypeptide 60	2,80	3,11E-06	C3H
Cyp2f2	cytochrome P450, family 2, subfamily f, polypeptide 2	2,79	2,35E-02	C3H
Itih2	inter-alpha trypsin inhibitor, heavy chain 2	2,78	4,17E-05	C3H
1500031L02Rik	RIKEN cDNA 1500031L02 gene	2,76	2,39E-12	C57BL/10
Gbp2	guanylate binding protein 2	2,71	3,99E-04	C3H
Zfp37	zinc finger protein 37	2,70	4,68E-05	C3H
Rcn1	reticulocalbin 1	2,70	5,09E-06	C3H
Retnlb	resistin like beta	2,69	2,42E-05	C3H
Bckdhb	branched chain ketoacid dehydrogenase E1, beta polypeptide	2,69	3,47E-10	C3H
Rbp2	retinol binding protein 2, cellular	2,68	2,31E-03	C3H
Pou2af1	POU domain, class 2, associating factor 1	2,67	5,33E-05	C3H

Table S1 (continued): Genes differentially expressed (fold change >2) between C57BL/10 and C3H mice

Gene Symbol	Gene Name	Fold change	FDR	Higher expressed in
Ahnak2	AHNAK nucleoprotein 2	2,67	3,67E-05	C57BL/10
Hoxb3	homeobox B3	2,64	3,89E-02	C57BL/10
Irgm2	immunity-related GTPase family M member 2	2,62	2,37E-06	C3H
Sval1	seminal vesicle antigen-like 1	2,62	6,87E-03	C3H
Abca12	ATP-binding cassette, sub-family A (ABC1), member 12	2,61	2,81E-02	C57BL/10
Zfp386	zinc finger protein 386 (Kruppel-like)	2,61	1,15E-09	C57BL/10
Prdm16	PR domain containing 16	2,60	1,20E-05	C57BL/10
Slc25a29	solute carrier family 25 (mitochondrial carrier, palmitoylcarnitine transporter), member 29	2,59	2,62E-11	C3H
Fig4	FIG4 homolog (S. cerevisiae)	2,55	8,49E-12	C57BL/10
Plcd3	phospholipase C, delta 3	2,55	1,87E-06	C57BL/10
Crym	crystallin, mu	2,54	7,44E-12	C3H
Ppp1r9a	protein phosphatase 1, regulatory (inhibitor) subunit 9A	2,53	3,14E-07	C57BL/10
Rgs13	regulator of G-protein signaling 13	2,53	1,78E-04	C57BL/10
H2-Q1	histocompatibility 2, Q region locus 1	2,51	8,64E-07	C3H
Ido1	indoleamine 2,3-dioxygenase 1	2,49	2,91E-02	C3H
Ikbip	IKBKB interacting protein	2,47	9,79E-08	C57BL/10
Ak4	adenylate kinase 4	2,46	1,29E-04	C57BL/10
Eif2c4	eukaryotic translation initiation factor 2C, 4	2,46	8,94E-09	C57BL/10
Timp3	tissue inhibitor of metalloproteinase 3	2,45	1,41E-03	C3H
Guca2b	guanylate cyclase activator 2b (retina)	2,44	2,16E-03	C3H
Ptk6	PTK6 protein tyrosine kinase 6	2,44	1,95E-05	C3H
Daam1	dishevelled associated activator of morphogenesis 1	2,43	6,84E-08	C3H
Plcxd2	phosphatidylinositol-specific phospholipase C, X domain containing 2	2,43	6,57E-09	C57BL/10
Pyroxd2	pyridine nucleotide-disulphide oxidoreductase domain 2	2,42	1,07E-07	C3H
Ifi27l1	interferon, alpha-inducible protein 27 like 1	2,41	4,33E-08	C3H
Plscr4	phospholipid scramblase 4	2,40	4,93E-06	C3H
Plbd1	phospholipase B domain containing 1	2,40	5,11E-05	C3H
Gbp3	guanylate binding protein 3	2,39	1,67E-05	C3H
Adh1	alcohol dehydrogenase 1 (class I)	2,39	1,32E-03	C3H
Mical2	microtubule associated monoxygenase, calponin and LIM domain containing 2	2,38	3,78E-08	C57BL/10
E030010A14Rik	RIKEN cDNA E030010A14 gene	2,36	6,13E-04	C57BL/10
NA	NA	2,35	5,05E-10	C57BL/10
Scara5	scavenger receptor class A, member 5 (putative)	2,34	3,24E-03	C57BL/10
Galnt2	UDP-N-acetyl-alpha-D-galactosamine:polypeptide N-acetylgalactosaminyltransferase 2	2,34	1,17E-08	C57BL/10

Table S1 (continued): Genes differentially expressed (fold change >2) between C57BL/10 and C3H mice

Gene Symbol	Gene Name	Fold change	FDR	Higher expressed in
Ift57	intraflagellar transport 57	2,34	9,85E-06	C3H
Trim30a	tripartite motif-containing 30A	2,33	3,82E-03	C3H
Efnb1	ephrin B1	2,31	9,66E-08	C57BL/10
Maob	monoamine oxidase B	2,31	1,56E-05	C3H
Wfdc2	WAP four-disulfide core domain 2	2,30	5,55E-03	C3H
Trim16	tripartite motif-containing 16	2,29	2,84E-07	C57BL/10
Fam40b	family with sequence similarity 40, member B	2,29	1,10E-08	C57BL/10
Glo1	glyoxalase 1	2,28	2,48E-13	C3H
Arsk	arylsulfatase K	2,28	3,78E-11	C57BL/10
Nipal1	NIPA-like domain containing 1	2,27	2,25E-05	C3H
Bcat2	branched chain aminotransferase 2, mitochondrial	2,26	4,46E-08	C3H
Spink4	serine peptidase inhibitor, Kazal type 4	2,25	1,47E-04	C57BL/10
Glycam1	glycosylation dependent cell adhesion molecule 1	2,25	3,58E-02	C3H
Gadd45g	growth arrest and DNA-damage-inducible 45 gamma	2,25	1,73E-03	C57BL/10
Scpep1	serine carboxypeptidase 1	2,25	7,80E-10	C3H
Qsox1	quiescin Q6 sulfhydryl oxidase 1	2,23	2,97E-04	C57BL/10
Gm12511	predicted gene 12511	2,23	1,19E-09	C57BL/10
Slc22a29	solute carrier family 22. member 29	2,21	1,20E-05	C3H
Nceh1	arylacetamide deacetylase-like 1	2,21	3,08E-03	C57BL/10
Hck	hemopoietic cell kinase	2,20	8,88E-05	C57BL/10
Tmem117	transmembrane protein 117	2,20	5,86E-08	C57BL/10
B130006D01Rik	RIKEN cDNA B130006D01 gene	2,20	1,25E-06	C57BL/10
Efemp1	epidermal growth factor-containing fibulin-like extracellular matrix protein 1	2,19	1,32E-03	C57BL/10
Sh2d4a	SH2 domain containing 4A	2,18	7,44E-12	C3H
Wfdc17	WAP four-disulfide core domain 17	2,18	2,27E-07	C57BL/10
2310034G01Rik	RIKEN cDNA 2310034G01 gene	2,18	1,03E-09	C3H
Sord	sorbitol dehydrogenase	2,17	2,27E-07	C57BL/10
Rps6kb1	ribosomal protein S6 kinase, polypeptide 1	2,17	3,25E-10	C57BL/10
Car8	carbonic anhydrase 8	2,17	2,20E-03	C57BL/10
Aim1l	absent in melanoma 1-like	2,17	2,82E-05	C57BL/10
Slc1a5	solute carrier family 1 (neutral amino acid transporter), member 5	2,16	4,20E-10	C57BL/10
Pfn1	profilin 1	2,16	1,44E-07	C3H
Slc38a1	solute carrier family 38, member 1	2,15	3,78E-11	C57BL/10
Irf9	interferon regulatory factor 9	2,15	9,79E-10	C3H

Table S1 (continued): Genes differentially expressed (fold change >2) between C57BL/10 and C3H mice

Gene Symbol	Gene Name	Fold change	FDR	Higher expressed in
Gulp1	GULP, engulfment adaptor PTB domain containing 1	2,14	3,77E-03	C57BL/10
Vegfc	vascular endothelial growth factor C	2,14	8,82E-06	C57BL/10
2010107G23Rik	RIKEN cDNA 2010107G23 gene	2,13	2,90E-12	C3H
Rassf4	Ras association (RalGDS/AF-6) domain family member 4	2,12	1,72E-04	C57BL/10
Ifitm3	interferon induced transmembrane protein 3	2,11	1,41E-04	C3H
Ttr	transthyretin	2,11	3,57E-02	C3H
Glt25d1	glycosyltransferase 25 domain containing 1	2,11	6,93E-10	C57BL/10
Lgals9	lectin, galactose binding, soluble 9	2,11	1,24E-05	C3H
Tspan6	tetraspanin 6	2,10	4,64E-04	C57BL/10
Dhx58	DEXH (Asp-Glu-X-His) box polypeptide 58	2,10	8,29E-05	C3H
F3	coagulation factor III	2,10	4,59E-02	C3H
Trmt61b	tRNA methyltransferase 61 homolog B (S. cerevisiae)	2,09	2,49E-09	C57BL/10
Eif4e3	eukaryotic translation initiation factor 4E member 3	2,09	2,06E-03	C57BL/10
Me1	malic enzyme 1, NADP(+)-dependent, cytosolic	2,09	2,77E-02	C57BL/10
Tesk2	testis-specific kinase 2	2,09	1,51E-06	C3H
Gas5	growth arrest specific 5	2,09	9,60E-06	C57BL/10
Gbp7	guanylate binding protein 7	2,07	1,67E-05	C3H
Sycn	syncollin	2,06	9,50E-03	C3H
Psmb9	proteasome (prosome, macropain) subunit, beta type 9 (large multifunctional peptidase 2)	2,06	9,49E-08	C3H
2200002K05Rik	RIKEN cDNA 2200002K05 gene	2,06	2,43E-04	C57BL/10
Car12	carbonic anyhydrase 12	2,06	7,68E-03	C57BL/10
Flywch2	FLYWCH family member 2	2,05	1,66E-05	C57BL/10
Ocel1	occludin/ELL domain containing 1	2,05	1,55E-11	C3H
Pyy	peptide YY	2,05	1,96E-04	C3H
Ly6a	lymphocyte antigen 6 complex, locus A	2,05	1,02E-02	C57BL/10
Acacb	acetyl-Coenzyme A carboxylase beta	2,04	2,16E-04	C57BL/10
Exd1	exonuclease 3'-5' domain containing 1	2,04	3,98E-11	C3H
Mul1	mitochondrial ubiquitin ligase activator of NFKB 1	2,04	2,75E-08	C57BL/10
Smek2	SMEK homolog 2, suppressor of mek1 (Dictyostelium)	2,04	1,81E-12	C3H
Cpn1	carboxypeptidase N, polypeptide 1	2,04	5,28E-03	C57BL/10
Tm6sf2	transmembrane 6 superfamily member 2	2,04	1,97E-03	C3H
Fam43a	family with sequence similarity 43, member A	2,02	1,86E-02	C57BL/10
Gadd45gip1	growth arrest and DNA-damage-inducible, gamma interacting protein 1	2,02	1,32E-08	C57BL/10
Thbs1	thrombospondin 1	2,02	3,19E-04	C3H

Table S1 (continued): Genes differentially expressed (fold change >2) between C57BL/10 and C3H mice

Gene Symbol	Gene Name	Fold change	FDR	Higher expressed in
Npc1	Niemann Pick type C1	2,02	4,63E-05	C57BL/10
Shoc2	soc-2 (suppressor of clear) homolog (C. elegans)	2,01	3,78E-10	C3H
Zmym6	zinc finger, MYM-type 6	2,01	2,85E-08	C57BL/10
Hsd17b13	hydroxysteroid (17-beta) dehydrogenase 13	2,01	2,76E-02	C57BL/10
Ly96	lymphocyte antigen 96	2,01	5,73E-07	C57BL/10
Afap1	actin filament associated protein 1	2,01	2,85E-05	C3H
