# Supplementary material for: Differences in Mucosal Gene Expression in the Colon of Two Inbred Mouse Strains after Colonization with Commensal Gut Bacteria
Source: PLoS One. 2013 Aug 9;8(8):e72317. doi: 10.1371/journal.pone.0072317 (PMC3739790; doi:10.1371/journal.pone.0072317)
Supplement: Table S3 — DAVID functional gene list: signaling/secretion. (PDF) [file pone.0072317.s003.pdf]

Table S3: DAVID functional gene list: signaling/secretion

Gene Symbol	Gene Name	Fold change	FDR	Higher expressed in
Pla2g2a	phospholipase A2, group IIA (platelets, synovial fluid)	70,80	1,62E-17	C3H
Ang4	angiogenin, ribonuclease A family, member 4	33,06	2,23E-07	C57BL/10
Slpi	secretory leukocyte peptidase inhibitor	28,44	5,23E-14	C3H
Qpct	glutaminyl-peptide cyclotransferase (glutaminyl cyclase)	18,57	1,04E-18	C3H
Pnliprp2	pancreatic lipase-related protein 2	15,17	2,19E-08	C57BL/10
Ppy	pancreatic polypeptide	10,39	8,83E-11	C3H
Apoc2	apolipoprotein C-II	8,96	1,97E-11	C3H
Mep1a	meprin 1 alpha	7,77	8,16E-13	C57BL/10
Afm	afamin	6,47	2,07E-15	C3H
Ly6g6c	lymphocyte antigen 6 complex, locus G6C	5,46	2,71E-07	C3H
Tff2	trefoil factor 2 (spasmolytic protein 1)	5,45	1,24E-04	C3H
Amn	amnionless	5,44	4,76E-09	C3H
P2ry6	pyrimidinergic receptor P2Y, G-protein coupled, 6	5,27	1,54E-12	C57BL/10
Tmem87a	transmembrane protein 87A	4,69	4,64E-15	C3H
1810030J14Rik	RIKEN cDNA 1810030J14 gene	4,66	7,66E-04	C57BL/10
Tlr1	toll-like receptor 1	4,34	8,16E-13	C57BL/10
Itln1	intelectin 1 (galactofuranose binding)	4,27	2,63E-02	C57BL/10
Cd14	CD14 antigen	4,17	1,11E-10	C3H
Ly6g	lymphocyte antigen 6 complex, locus G	4,11	4,30E-03	C3H
Tns4	tensin 4	3,95	2,68E-08	C57BL/10
Clps	colipase, pancreatic	3,79	2,07E-06	C57BL/10
Cd74	CD74 antigen (invariant polypeptide of major histocompatibility complex, class II antigen-associated)	3,61	2,27E-07	C3H
Col8a1	collagen, type VIII, alpha 1	3,46	5,90E-04	C3H
Insl5	insulin-like 5	3,44	1,74E-03	C3H
H2-Bl	histocompatibility 2, blastocyst	3,20	8,76E-08	C3H
Defb37	defensin beta 37	3,13	1,86E-03	C57BL/10
Crim1	cysteine rich transmembrane BMP regulator 1 (chordin like)	2,98	6,90E-11	C57BL/10
Ermap	erythroblast membrane-associated protein	2,97	3,57E-03	C57BL/10
Apol6	apolipoprotein L 6	2,90	2,19E-06	C3H
Lyz2	lysozyme 2	2,87	1,10E-05	C3H
Tor3a	torsin family 3, member A	2,86	3,78E-10	C3H
Ptprf	protein tyrosine phosphatase, receptor type, F	2,85	1,20E-10	C3H
Itih2	inter-alpha trypsin inhibitor, heavy chain 2	2,78	4,17E-05	C3H
Rcn1	reticulocalbin 1	2,70	5,09E-06	C3H

Table S2: DAVID functional gene list: signaling/secretion

Gene Symbol	Gene Name	Fold change	FDR	Higher expressed in
Retnlb	resistin like beta	2,69	2,42E-05	C3H
Sval1	seminal vesicle antigen-like 1	2,62	6,87E-03	C3H
H2-Q1	histocompatibility 2, Q region locus 1	2,51	8,64E-07	C3H
Timp3	tissue inhibitor of metalloproteinase 3	2,45	1,41E-03	C3H
Guca2b	guanylate cyclase activator 2b (retina)	2,44	2,16E-03	C3H
Plbd1	phospholipase B domain containing 1	2,40	5,11E-05	C3H
Scara5	scavenger receptor class A, member 5 (putative)	2,34	3,24E-03	C57BL/10
Galnt2	UDP-N-acetyl-alpha-D-galactosamine:polypeptide N-acetylgalactosaminyltransferase 2	2,34	1,17E-08	C57BL/10
Efnb1	ephrin B1	2,31	9,66E-08	C57BL/10
Wfdc2	WAP four-disulfide core domain 2	2,30	5,55E-03	C3H
Arsk	arylsulfatase K	2,28	3,78E-11	C57BL/10
Spink4	serine peptidase inhibitor, Kazal type 4	2,25	1,47E-04	C57BL/10
Glycam1	glycosylation dependent cell adhesion molecule 1	2,25	3,58E-02	C3H
Scpep1	serine carboxypeptidase 1	2,25	7,80E-10	C3H
Qsox1	quiescin Q6 sulfhydryl oxidase 1	2,23	2,97E-04	C57BL/10
Efemp1	epidermal growth factor-containing fibulin-like extracellular matrix protein 1	2,19	1,32E-03	C57BL/10
Pfn1	profilin 1	2,16	1,44E-07	C3H
Vegfc	vascular endothelial growth factor C	2,14	8,82E-06	C57BL/10
Ttr	transthyretin	2,11	3,57E-02	C3H
Glt25d1	glycosyltransferase 25 domain containing 1	2,11	6,93E-10	C57BL/10
Lgals9	lectin, galactose binding, soluble 9	2,11	1,24E-05	C3H
F3	coagulation factor III	2,10	4,59E-02	C3H
Sycn	syncollin	2,06	9,50E-03	C3H
2200002K05Rik	RIKEN cDNA 2200002K05 gene	2,06	2,43E-04	C57BL/10
Car12	carbonic anyhydrase 12	2,06	7,68E-03	C57BL/10
Pyy	peptide YY	2,05	1,96E-04	C3H
Ly6a	lymphocyte antigen 6 complex, locus A	2,05	1,02E-02	C57BL/10
Cpn1	carboxypeptidase N, polypeptide 1	2,04	5,28E-03	C57BL/10
Thbs1	thrombospondin 1	2,02	3,19E-04	C3H
Npc1	Niemann Pick type C1	2,02	4,63E-05	C57BL/10
Hsd17b13	hydroxysteroid (17-beta) dehydrogenase 13	2,01	2,76E-02	C57BL/10
Ly96	lymphocyte antigen 96	2,01	5,73E-07	C57BL/10
