# Supplementary material for: Differences in Mucosal Gene Expression in the Colon of Two Inbred Mouse Strains after Colonization with Commensal Gut Bacteria
Source: PLoS One. 2013 Aug 9;8(8):e72317. doi: 10.1371/journal.pone.0072317 (PMC3739790; doi:10.1371/journal.pone.0072317)
Supplement: Table S4 — DAVID functional gene list: peptidase inhibition. (PDF) [file pone.0072317.s004.pdf]

Table S4: DAVID functional gene list: peptidase inhibition

Gene Symbol	Gene Name	Fold change	FDR	Higher expressed in
Slpi	secretory leukocyte peptidase inhibitor	28,44	5,23E-14	C3H
Crim1	cysteine rich transmembrane BMP regulator 1 (chordin like)	2,98	6,90E-11	C57BL/10
Itih2	inter-alpha trypsin inhibitor, heavy chain 2	2,78	4,17E-05	C3H
Timp3	tissue inhibitor of metalloproteinase 3	2,45	1,41E-03	C3H
Wfdc2	WAP four-disulfide core domain 2	2,30	5,55E-03	C3H
Spink4	serine peptidase inhibitor, Kazal type 4	2,25	1,47E-04	C57BL/10
Wfdc17	WAP four-disulfide core domain 17	2,18	2,27E-07	C57BL/10
