# Supplementary material for: Differences in Mucosal Gene Expression in the Colon of Two Inbred Mouse Strains after Colonization with Commensal Gut Bacteria
Source: PLoS One. 2013 Aug 9;8(8):e72317. doi: 10.1371/journal.pone.0072317 (PMC3739790; doi:10.1371/journal.pone.0072317)
Supplement: Table S5 — DAVID functional gene list: guanine binding. (PDF) [file pone.0072317.s005.pdf]

Table S5: DAVID functional gene list: Guanine binding

Gene Symbol	Gene Name	Fold change	FDR	Higher expressed in
Gbp1	guanylate binding protein 1	18,38	4,77E-16	C3H
Gbp2	guanylate binding protein 2	2,71	3,99E-04	C3H
Irgm2	immunity-related GTPase family M member 2	2,62	2,37E-06	C3H
Ak4	adenylate kinase 4	2,46	1,29E-04	C57BL/10
Gbp3	guanylate binding protein 3	2,39	1,67E-05	C3H
Gbp7	guanylate binding protein 7	2,07	1,67E-05	C3H
