# Supplementary material for: Differences in Mucosal Gene Expression in the Colon of Two Inbred Mouse Strains after Colonization with Commensal Gut Bacteria
Source: PLoS One. 2013 Aug 9;8(8):e72317. doi: 10.1371/journal.pone.0072317 (PMC3739790; doi:10.1371/journal.pone.0072317)
Supplement: Table S6 — DAVID functional gene list: response to bacteria/defense. (PDF) [file pone.0072317.s006.pdf]

## Table S6: DAVID functional gene list: response to bacteria/defense

| Gene Symbol | Gene Name | Fold change | FDR | Higher expressed in |
|---|---|---|---|---|
| Ang4 | angiogenin, ribonuclease A family, member 4 | 33,06 | 2,23E-07 | C57BL/10 |
| Pnliprp2 | pancreatic lipase-related protein 2 | 15,17 | 2,19E-08 | C57BL/10 |
| Tlr1 | toll-like receptor 1 | 4,34 | 8,16E-13 | C57BL/10 |
| Cd74 | CD74 antigen (invariant polypeptide of major histocompatibility complex, class II antigen-associated) | 3,61 | 2,27E-07 | C3H |
| Defb37 | defensin beta 37 | 3,13 | 1,86E-03 | C57BL/10 |
| Fkbp1b | FK506 binding protein 1b | 2,94 | 2,00E-06 | C3H |
| Lyz2 | lysozyme 2 | 2,87 | 1,10E-05 | C3H |
| H2-Q1 | histocompatibility 2, Q region locus 1 | 2,51 | 8,64E-07 | C3H |
| Ido1 | indoleamine 2,3-dioxygenase 1 | 2,49 | 2,91E-02 | C3H |
| Timp3 | tissue inhibitor of metalloproteinase 3 | 2,45 | 1,41E-03 | C3H |
| Adh1 | alcohol dehydrogenase 1 (class I) | 2,39 | 1,32E-03 | C3H |
| Hck | hemopoietic cell kinase | 2,20 | 8,88E-05 | C57BL/10 |
| Dhx58 | DEXH (Asp-Glu-X-His) box polypeptide 58 | 2,10 | 8,29E-05 | C3H |
| F3 | coagulation factor III | 2,10 | 4,59E-02 | C3H |
| Me1 | malic enzyme 1, NADP(+)-dependent, cytosolic | 2,09 | 2,77E-02 | C57BL/10 |
| Thbs1 | thrombospondin 1 | 2,02 | 3,19E-04 | C3H |
| Ly96 | lymphocyte antigen 96 | 2,01 | 5,73E-07 | C57BL/10 |
