# Supplementary material for: Differences in Mucosal Gene Expression in the Colon of Two Inbred Mouse Strains after Colonization with Commensal Gut Bacteria
Source: PLoS One. 2013 Aug 9;8(8):e72317. doi: 10.1371/journal.pone.0072317 (PMC3739790; doi:10.1371/journal.pone.0072317)
Supplement: Table S7 — DAVID functional gene list: hormone activity. (PDF) [file pone.0072317.s007.pdf]

Table S7: DAVID functional gene list: hormone activity

Gene Symbol	Gene Name	Fold change	FDR	Higher expressed in
Ppy	pancreatic polypeptide	10,39	8,83E-11	C3H
Tff2	trefoil factor 2 (spasmolytic protein 1)	5,45	1,24E-04	C3H
Insl5	insulin-like 5	3,44	1,74E-03	C3H
Retnlb	resistin like beta	2,69	2,42E-05	C3H
Vegfc	vascular endothelial growth factor C	2,14	8,82E-06	C57BL/10
Ttr	transthyretin	2,11	3,57E-02	C3H
Pyy	peptide YY	2,05	1,96E-04	C3H
