# Supplementary material for: Differences in Mucosal Gene Expression in the Colon of Two Inbred Mouse Strains after Colonization with Commensal Gut Bacteria
Source: PLoS One. 2013 Aug 9;8(8):e72317. doi: 10.1371/journal.pone.0072317 (PMC3739790; doi:10.1371/journal.pone.0072317)
Supplement: Table S8 — DAVID functional gene list: lipoprotein-associated. (PDF) [file pone.0072317.s008.pdf]

Table S8: DAVID functional gene list: lipoprotein-associated

Gene Symbol	Gene Name	Fold change	FDR	Higher expressed in
Gbp1	guanylate binding protein 1	18,38	4,77E-16	C3H
Plscr2	phospholipid scramblase 2	16,62	3,11E-14	C3H
Apoc2	apolipoprotein C-II	8,96	1,97E-11	C3H
Mal	myelin and lymphocyte protein, T cell differentiation protein	7,13	6,05E-07	C3H
Ly6g6c	lymphocyte antigen 6 complex, locus G6C	5,46	2,71E-07	C3H
Itln1	intelectin 1 (galactofuranose binding)	4,27	2,63E-02	C57BL/10
Cd14	CD14 antigen	4,17	1,11E-10	C3H
Ly6g	lymphocyte antigen 6 complex, locus G	4,11	4,30E-03	C3H
Apol6	apolipoprotein L 6	2,90	2,19E-06	C3H
Gbp2	guanylate binding protein 2	2,71	3,99E-04	C3H
Plscr4	phospholipid scramblase 4	2,40	4,93E-06	C3H
Hck	hemopoietic cell kinase	2,20	8,88E-05	C57BL/10
F3	coagulation factor III	2,10	4,59E-02	C3H
Ly6a	lymphocyte antigen 6 complex, locus A	2,05	1,02E-02	C57BL/10
